# Lessons learned from ten years of the Mountain West Clinical and Translational Research Infrastructure Network: A case study from the research core

**DOI:** 10.1017/cts.2025.10081

**Published:** 2025-07-24

**Authors:** Kathrene R. Conway, Lorraine S. Evangelista, Curtis W. Noonan, Jeffrey L. Ebersole, Reimund Serafica, Joseph Guerrero Lopez, Robert Scott Seville, Jay Shen, Francisco S. Sy

**Affiliations:** 1 University of Nevada Las Vegas, Las Vegas, NV, USA; 2 University of Montana, Missoula, MT, USA; 3 Louise Herrington School of Nursing, Baylor University, Dallas, TX, USA; 4 University of Wyoming, Laramie, WY, USA

**Keywords:** Translational research centers, core support, application process, review process, lessons learned

## Abstract

The Mountain West Clinical & Translational Research Infrastructure Network (MW CTR-IN), hosted at the University of Nevada, Las Vegas, and supported by the National Institute of General Medical Sciences at the National Institutes of Health, began as a partnership among 13 major U.S. public universities across 7 MW Institutional Development Award (IDeA) States, stretched across 1/3rd of the U.S. land mass and encompassed almost 1/3^rd^ of all IDeA States. The mission of the MW CTR-IN is to build and enhance infrastructure capacity to increase CTR in the MW region. This case study describes the Clinical Pilot Projects Program (CP3) processes and tools used to support this objective through its selection of applications to fund, supporting applicants and funded investigators, and providing guidance and oversight during the funding period. The MW CTR-IN has funded 152 single-investigator pilot projects, 7 multisite pilot projects, 13 developmental team grant projects, and 14 community-engaged projects. These projects have also led to over $92M in extramural grant funding, 308 presentations, and 1,124 peer-reviewed publications. The methodologies and expertise we gained can assist other CTR networks in developing efficient pilot project programs that have been evaluated and demonstrated to improve CTR initiatives, especially through the use of a custom portal.

## Overview

U.S. Populations that have historically had lower levels of NIH funding are less likely to have access to clinical research that addresses health conditions and health disparities of greatest concern in these states. The Institutional Development Award (IDeA), administered by the National Institute of General Medical Sciences (NIGMS), is a congressionally mandated program that builds research capacity in 24 states and Puerto Rico [1,2]. The IDeA-supported MW CTR-IN Program, established in 2013, was a partnership of 13 universities across 7 states to develop a culture of collaborative multidisciplinary CTR within and across western IDeA institutions to positively impact health disparities in our communities. The IDeA program incorporates states with proportionately large rural and medically underserved populations [1]. During FY01-10:

- 6/7 partner states ranked in the top 8 for lowest population density [2].
- 4/7 partner states (MT, AK, ID, WY) have >34% of their populations living in rural areas compared to the national average of 21% [3].
- 3/7 partner states have highest proportions of American Indian/Alaska Native populations (AK, NM, MT),
- 1 state has the highest proportion of Native Hawaiian/Pacific Islander populations (HI) [4],
- 2/7 partner states are among the top 5 in the US for proportion of population that identifies as Hispanic or Latino (NM, NV) [4]. Even in states with lower overall proportions of Hispanic populations (MT, ID, WY, AK), this demographic in the years 2000–2015, Hispanic populations increased 76%–86% compared to non-Hispanic population increases of 11%–20% [3,5,6].

This case study outlines how the MW CTR-IN’s Clinical Pilot Projects Program Core (CP3-C) facilitated a successful program across a wide region with different health needs. Most importantly, pilot project initiatives catalyzed and accelerated CTR from bench to bedside to improve health for the regional Mountain West communities [7].

**Figure 1.**
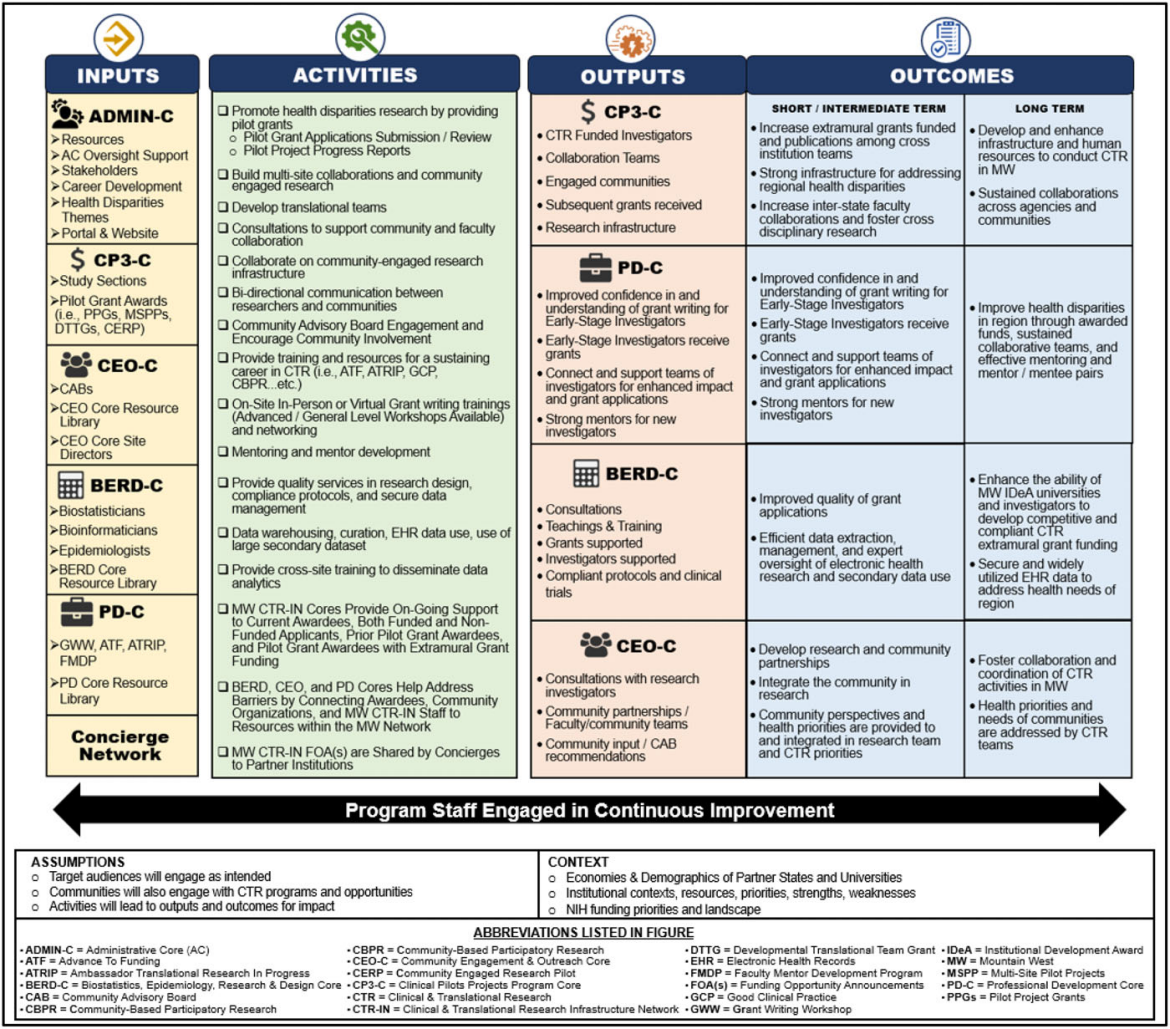
Is a logic model that summarizes the active core interactions with the CP3 core and its outcomes. This model provides a structure to monitor program impact and guide on-going refinement, especially in the development of our custom portal. The alignment between planned outcomes and observed impacts supports the model’s validity as a tool for managing translational research programs in underserved regions.

We present our perspectives on the experience gained from lessons learned over the past decade and the strategies employed to surmount problems by implementing new processes to optimize CP3 activities. We will highlight the collaboration between the CP3-C and the Network’s five additional cores—Administrative Core (Admin-C), Professional Development Core (PD-C), Community Engagement & Outreach Core (CEO-C), Biostatistics, Epidemiology, Research & Design Core (BERD-C), and Tracking & Evaluation Core (T&E-C) to enhance the implementation of best practices within the CP3 initiative; however, the focus will be upon the CP3-C. Figure 1 summarizes the cross-core interactions among the CP3-C and the other Cores’ activities and outcomes.

**Figure 2.**
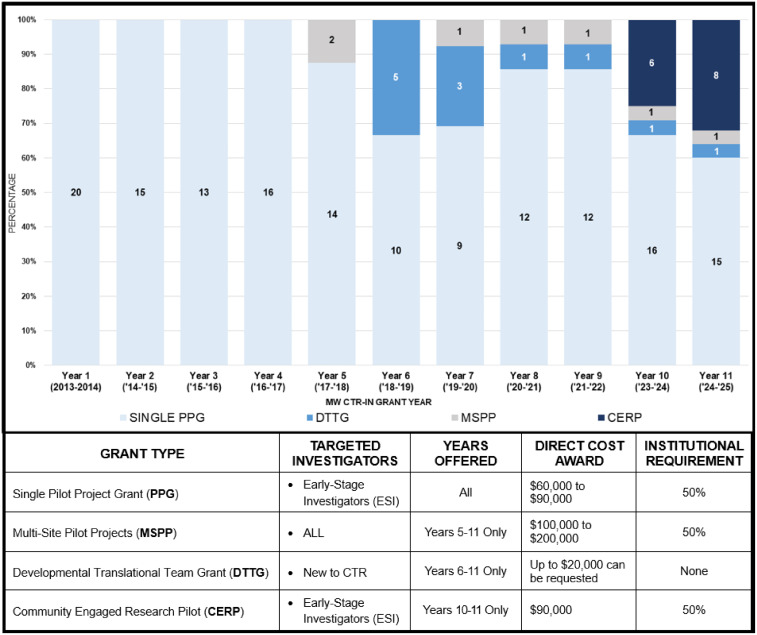
Illustrates the pilot grant awards for each mechanism by year. Single pilot project grants (PPG) awards were awarded from years 1–11. While new award mechanisms such as the multisite pilot project (MSPP), developmental translational team grants (DTTG) and community engaged research pilot (CERP) awards were only added on years 5, 6, and 10 respectively.

**Figure 3.**
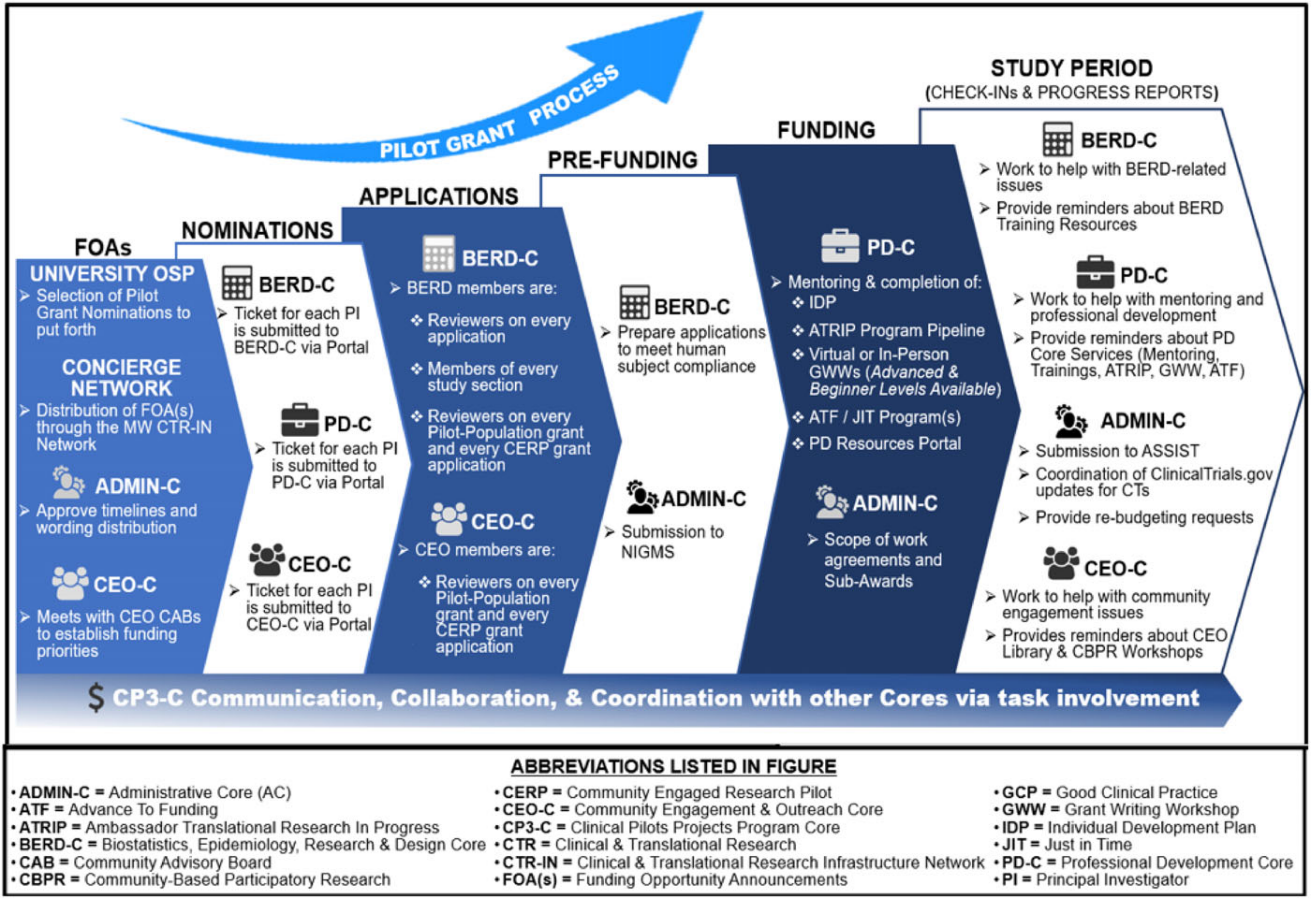
The CP3 core communicates, collaborates and coordinates with other cores as grant applications are reviewed and awardees are selected throughout the MW CTR-IN pilot grant process.

The MW CTR-IN and CP3-C developed a pilot program to solicit, review, award, and monitor PGs to support NIH defined New and/or Early Stage Investigators (ESIs) in establishing nationally competitive independent research careers. Over the entire grant program, New Investigators (both early and non-early) accounted for 86% of applicants and 99% of funded PGs. Though the majority of the focus was on ESIs, there was also a need to assist mid-career scientists with developing existing bench research programs into CTR and to provide support towards the formation of new multidisciplinary teams to effectively compete in new research funding areas. To support the efforts of the mid-career faculty, the CTR-IN added a Multi-Site Pilot Projects (MSPPs) and Developmental Translational Team Grants (DTTGs). In the last few years, a Community-Engaged Research Pilot (CERP) mechanism was added to support existing community-engaged teams. Figure 2 details each of the MW CTR-IN’s grant mechanisms given over the past 10 years. MW CTR-IN PG awardees engaged in cutting-edge, early-stage pilot projects or explored novel, high-risk research avenues throughout the clinical translational research spectrum (T1–T4) [8,9]. The 117 unique investigators have led to over $92M in extramural grant funding from 44 extramural grants, 308 presentations, and 1,124 peer-reviewed publications. These grants spanned all institutions (range of 1–26, median of 8.16, and mode of 7). This success was aided greatly by the institutional support of the research offices, the concierge network, and other CTR-IN Cores as seen in Figure 3.

## Institutional support

Each institution supported the network in important ways. First, as part of the CTR consortium, all institutions agreed to a 10% Facilities and Administration (F&A) cost on PG funds allocated to investigators. Given the many institutions involved, this commitment was crucial for ensuring that sufficient direct cost budget resources flowed to investigators. As appropriate, the full F&A rate was charged to CTR-IN personnel at each institution (e.g., biostatisticians). Pilot applications required that the PI devote at least 20% effort to the research activity, but only 50% of the total PI effort was charged as direct cost to the pilot award budget. Per prior agreement, the balance of PI effort was covered by institutional support in the form of release from teaching, assignment of time provided to pursue scholarly activity, or other mechanisms appropriate to the institution. Applications required a letter of commitment from an administrative official (i.e., department chair or dean), but this support was not considered formal cost share nor did CTR-IN require corresponding reporting on this commitment. Each institution provided a concierge who served as the key communication pathway for notices of funding opportunities and associated grant application trainings (see below). Finally, each institution’s Vice President for Research (VPR) or delegate served on the Internal Advisory Committee (IAC) whose role is discussed in the review process section.

## Concierge network (CN)

Each partner university identified a faculty or administrator to serve as an unpaid MW CTR-IN CN member. Concierges disseminated the pilot Notices of Funding Opportunities (NOFOs) within their institutions and helped to connect prospective faculty investigators with former award recipients, Office of Sponsored Programs (OSP) representatives, and other research support resources at their institution. Monthly virtual CN meetings led by the ADMIN-C and the CP3-C CN Chair ensured faculty had the requisite information to advocate for the MW CTR-IN at each campus. The CN enabled broad distribution of the program NOFOs, resulting in grant applications received from multiple disciplines such as medicine, public health, nursing, allied health, biological and life sciences, education, kinesiology, nutrition, humanities, computer science, mechanical engineering, microbiology, physics, and mathematics- many of which are not traditionally considered to be CTR departments.

## MW CTR-IN cores

Prospective applicants were urged to leverage the Program’s research support service cores (BERD-C, PD-C, and CEO-C). Figure 3 shows how each of the cores were engaged throughout the lifecycle of the projects. BERD-C consultations were assigned to pilot project candidates developing methodological and data analysis plans for their award submissions. The core personnel, including epidemiologists, biostatisticians, and bioinformaticians, continued supporting the funded applicants with biostatistical analysis, MSPP Virtual Workshops, and contributions to subsequent extramural grant submissions. The PD-C members were involved in the application process by (if needed) matching pilot investigators with senior scientific mentors [10], providing training opportunities and pre-submission critiques of their applications. After funding, the PD-C provided in-person/virtual grant writing workshops (GWW), mock grant reviews of external grant applications before external submissions, and virtual monthly meetings with didactic sessions through a mentor-peer network to discuss their work in progress. The CEO-C Site Directors provided support by offering culturally appropriate research services and consultations to advance research concepts focused on vulnerable or marginalized populations, aid in disseminating research findings to enhance the reach and impact of these studies, and the development of a community-engaged resource library of publications and trainings.

## Institutional review board (IRB) reliance

Through the efforts of the University of New Mexico – Health Sciences Center, a MW Research Consortium Common Reciprocal IRB Reliance Authorization Agreement was signed, which allowed the Lead Site PI university to be the IRB of record. Half of our multisite projects were able to use this agreement, which did not cover indigenous communities.

## The review and selection process

Each grant application underwent a rigorous review by one of several Scientific Review Groups (SRG) composed of scholars from various professional areas. Each SRG utilized a review process similar to NIH. The primary and secondary reviewers were typically senior investigators with expertise in the sciences pertinent to the application, with a BERD-C member also providing review. The SRG review process and scoring form are structured based on the standard NIH review criteria: “Significance,” “Investigators,” “Innovation,” “Approach,” and “Environment.” Reviewers were provided with the Notice of Funding Opportunities (NOFO), which included the current CTR-IN funding priorities. These scoring criteria, funding priorities, and the reviewer’s assessment of the likelihood that the pilot research would generate the key preliminary data necessary for a subsequent extramural grant application contributed to the Overall Impact score. Regardless of the initial average impact score, all applications were discussed during the SRG to maximize critical feedback for applicants.

The rankings of the final overall impact scores were presented by CP3 for discussion by the advisory committees. The Internal Advisory Committee (IAC) (composed of the Vice Presidents for Research or equivalent, at each partner university) provided essential feedback to guide the distribution of awards across the consortium. The External Advisory Committee (EAC), comprising of 6 to 8 external/well-established leaders/senior research investigators, provided essential feedback on the scientific merit of proposals based on the written critiques. Each group made funding recommendations to the MW CTR-IN leadership and its PI, who made final funding recommendations. Those selected were forwarded to NIGMS for approval.

## Engaging the community in the review and selection process

In 2018, the CEO-C was established to empower and engage the community in CTR within their respective geographic regions. This initiative was founded on the premise that community members possess unique insights into their communities’ health priorities and needs, thereby bolstering the capacity-building efforts of academic-community partnerships to undertake community-engaged research, ultimately fostering transformative changes and better health outcomes [12]. Community Advisory Boards were formed within each region of the CTR-IN which resulted in a list of funding priorities for the NOFOs.

CEO-C members were reviewers of CERP applications to facilitate a collaborative approach and enhance the balance between community and academic perspectives.

## Discussion

Over the decade-long existence of the MW CTR-IN, several revisions have occurred in the application and review processes to establish a network that finances significant CTR while fostering the development of funded investigators. Our decade of experience has revealed numerous challenges in how we operationalized the CP3 initiative and addressed challenges that can inform the establishment of new centers.

### Portal development

Of the 13 CTR-Ns that have been funded, 7 of them exist within a single state. Of the 6 that involve multiple states, 3 are comprised of 2 states, 1 has 3 states, and 1 has 4 states [11]. With 13 separate institutions across 7 states and 3 time zones, a system was needed to provide a single-point of entry for all data needed to support investigators. Various levels of access to the data within each institution necessitated a data management strategy that was robust and flexible. The MW CTR-IN invested in a custom portal using Laravel, PHP, and MySQL. The portal quickly evolved into over 120 tables, 500 forms, and 10 distinct sections to support the application submission and review process, track the progress of funded projects and capture the information needed for reporting, provide core resources to funded/non-funded investigators, and capture metrics for tracking and evaluation.

Various layers of portal access were created based on designated roles within the center. Core leadership could see all the data across the entire portal, but VPRs could only see the data for their institution. For added security, registration in the portal was restricted to those who had an email domain from partner institutions. However, special registration invites allowed access to specific portions for community partners, advisory committee members, and reviewers.

Investigators can submit applications, provide just-in-time documentation, receive funding decisions, access reviews, create support tickets for core services, enter progress reports, access the libraries and training resources, develop Individual Development Plans (IDP), and/or participate in core programs like GWW(s) and didactic monthly meetings.

BERD, CEO, and PD Cores were able to see the pilot applications and PI information; view support tickets assigned to them; document interactions with investigators, view metrics regarding tickets & interactions; and update the core’s sections of the portal. All cores had their own library for resources/trainings and could control the content within them. When resources were viewed, tracking functionality recorded the user, resource, and date/time of viewing. BERD-C members submitted quarterly productivity reports. The PD-C used the portal for managing GWW(s), pre-reviews of external grant submissions, and monthly didactic meetings. T&E-C can measure investigator to center “touches” and evaluate the center’s impact on careers. T&E-C can download raw data, view summarized usage and metric tables, and configure automated evaluation links after triggering events.

The system administration, stock emails, and automation options allow the cores to control phases and rounds of applications, create stock emails with piped data, and to send reminders on schedules. The advantage of using these mechanisms is that programming changes and technological knowledge are not needed to update the portal. Additional information about the portal related to CP3 functions will be discussed in the grant lifecycle sections below.

### During the application process

*Developing and utilizing the portal to complete and submit grant applications*. During the program’s initial years, most of the application process was conducted via email and directly between the PI and the CP3-C. To overcome the challenges posed by disseminating and receiving multiple documents via email, we established a centralized portal for PG application submissions, reviews, and communication with applicants. While many other grant centers use REDCap for the application process, access to this resource is typically available through CTSA centers. With the exception of one partner, the CTR-IN institutions did not have CTSAs, and managing the vast number of consortium investigators who needed varying levels of access rendered REDCap a non-viable option for this use case. Our portal solved both of these problems and also provided the added benefit of ensuring that all information was up-to-date, accessible to only those who should have access, properly recorded, easily connected to all of the other sections of the portal, and automatically captured in metric tables,

*Limiting the number of applications from each university.* In the award’s inaugural year, an overwhelming 142 letters of intent (LOI) were received. The submissions received demonstrated a wide range of scientific quality and grant-writing skills across the 13 institutions. In response, the process changed to require university OSP offices or delegates to submit a maximum number of LOIs - typically 4 per mechanism. Each university had internal review processes to nominate application based on the institution’s priorities. Delegating the responsibility of soliciting and providing a preliminary selection of submissions resulted in improved quality of applications and assurance of institutional support.

*Using the letter of intent to identify potential issues.* The letter of intent stage enabled the CP3-C to screen for projects or applicants that would not be eligible for funding under specific funding mechanisms. Investigators could adjust the scope or institutions were able to replace the non-eligible projects.

*Engaging the Office of Sponsored Programs (OSP)*. Initially, pilot project applicants generally did not confer with or seek guidance from their institutions’ OSP before submitting their own applications. This lack of reliance on the OSP’s experience with grant application specifications, budget preparation, and federal requirements for human subjects research led to numerous incomplete applications and applications that did not follow the NOFO guidelines. OSP offices received copies of the NOFOs and Application Instructions and the OSP submission of the applications yielded improved quality of applications and fewer non-compliant budgetary and human subjects components.

*Automatically issuing tickets to provide coordinated and synergistic support from the service cores.* We attempted several methods to encourage applicants to engage with the service cores (PD-C, BERD-C, and CEO-C), but we had limited success in encouraging applicants to engage without it being a requirement for submission, and requiring contact for submission often was met last minute with no time for meaningful support. Our successful strategy was to utilize the portal to automatically generate tickets when the invitation to submit a full proposal was sent. The tickets linked each applicant with their designated support contact from each service core. Core team members contacted each applicant to introduce themselves, outline their core services, assess support needs, and record these interactions in the portal. Core team members could also add other team members to the ticket when specific areas of expertise were needed.

*Designing a mechanism for ad hoc core support.* We also developed a portal feature allowing funded pilot investigators, previously funded investigators, or current applicants to submit an ad hoc request for core services. This request generated tickets for each core team member responsible for its execution. This process ensured rapid response to support requests and allowed for documenting and collating core utilization.

*Documenting essential interactions with applicants or pilot awardees.* In any institution, personnel turnover is inevitable. Without documentation regarding the interactions between core team members and investigators, transferring that knowledge from one former support core team member to the next becomes exceedingly difficult. Mandating all team members to log their interactions with investigators allowed new members to review the historical records. Furthermore, it enabled the cores to function collaboratively rather than in isolation, facilitating comprehensive visibility of all interactions with an investigator across the entire network. The pilot investigator could also access these notes, ensuring accountability and transparency. Through the years of implementation, 558 tickets were created, resulting in 1,510 logged interactions.

*MSPPs.* Initially, the MSPPs were required to involve multiple states and at least three universities. CTR-IN leadership had anticipated that cross-institutional collaborations would develop organically; however, applications to this mechanism remained low. CTR-IN personnel attempted to develop interest groups on particular content areas that were well represented across the network. However, the feasibility of establishing such partnerships with these requirements proved detrimental to application submissions, and this requirement was eventually modified to a minimum of any two universities. Further work on how to establish multi-state clinical research partnerships in these IDeA states is needed, and some evidence of success has been established elsewhere through the IDeA States Pediatric Clinical Trials Network.

*DTTG.* Requiring the universities to support the 50% of the effort of the investigators ensured that investigators were supported and had time to complete the project. A 50% match by the university for physicians and dentists created a barrier to these investigators being able to apply for our other mechanisms. Because this mechanism did not require an institutional match, practicing clinicians could apply and engage in research.

In 2022, in support of NIH’s strategic initiative to enhance capacity-building and assist investigators and their already established partners in conducting community-engaged research, a funding opportunity for the Community-Engaged Research Pilot (CERP) was created. Applications for the CERP were required to have an established community partnership which could be built upon to conduct the proposed research. The details of the partner’s engagement are detailed in the application, outlined in the partners Letter of Support, and included in the project both budgetarily (at least 5% had to go to the community partner) and administratively. Applicants highlighted the project’s addressing of community-identified funding priorities in their significance sections.

### During the review process

*Utilizing the portal throughout the review process.* During the MW CTR-IN’s initial years, review templates and application files were emailed to reviewers who provided feedback via email. This process caused frequent delays, increased the likelihood of data entry errors, required additional administrative hours, and warranted a change to streamline the process. We developed portal features to support scientific merit reviews similar to the Internet Assisted Review feature in eRA Commons. Reviewers receive access to the applications, instructions and review templates and submit reviews and overall impact scores in the portal. Automated reminders were sent one week and one day before deadlines. The portal also allowed for real-time submission of final impact scores by panel members following discussion.

*Employing an algorithm matching applications and reviewers.* Over the years of the program, we curated a pool of over 150 reviewers. Reviewers indicated their areas of expertise when they registered in the portal. During application submission, three areas of expertise were entered to guide our reviewer-matching process. Our existing reviewer pool annually is prompted to update in the portal their information and availability among the potentials dates for panels. Typically, 40%–50% are update annually. Gaps in expertise are filled by core leaders recruiting new reviewers via registration invitations.

Using reviewer availability and translational areas (e.g., Bench, Clinical, Population), a date for each panel is selected. Reviewers matching availability and expertise are assigned to panels. After submission, applications are assigned to study sections based on their translational area. For each application, a score is automatically computed for each reviewer in the assigned panel. (A first topic area match is 20 points; the second and third matches are worth 10 and 5 points, respectively.) Reviewers not at the same institution were assigned by highest score matches. Same institution SRG members are recused during the application’s panel discussion.

*All reviewers are required to revise reviews after the SRG review session.* Given that most MW CTR-IN applicants are New or early-stage researchers, our goal is not only for the reviews to yield scores that facilitate funding decisions but also for mentorship. Consequently, we require all members of the SRG to reassess their written reviews after the panel discussion to guarantee that all score-influencing aspects are thoroughly noted. We consistently highlighted that these reviews aim to guide new and early career investigators in enhancing their pilot projects, regardless of funding status.

For the CTR-IN, the funding scores are not the only consideration for funding. After completion of the SRGs, applications are ranked by score, score within study section, and score within institution. The CP3-C facilitates two committee meetings to put forth recommendations for funding to the Network PI. The EAC is primary focused on funding the best science, while the IAC focuses on both funding the best science and ensuring that, where justifiable, all participating universities received some funding. Tables are calculated in the portal to show number of submission per institution per mechanism, number of applications eligible for funding per mechanism per institution if the cut-off for funding is an Overall Impact Score of 40, the same information if the cut-off is 50, and a view that shows the best score received by an institution for each funding mechanism. The EAC and IAC provide a letter to the Network PI with their recommendations for funding and the additional applications that they support funding if additional funds are available. In some cases, the EAC would agree to funding some lower scoring applications if an A1 revision adequately addressed the inadequacies identified in the reviews.

### During the funding cycle

*Requiring the creation of a professional development plan during the grant funding year*. Many of our investigators had substantial responsibilities, more broadly than research, in their early careers, especially after being awarded their first pilot project. Thus, part of the Scope of Work issued with the award instructed applicants to work with their PD-C team members and mentor to complete an Individual Development Plan (IDP). The IDP included goals, timelines for completing publications and grant applications relevant to the funded pilot award.

*Quarterly progress reports.* These were also entered into the portal and allowed the CP3 to intervene early if there were recruitment issues. This information was used by Admin core to update the Assist program.

## Conclusion

After ten years of experience, the application, funding, and PI support processes are simpler and more efficient as evidenced by how our logic model outcomes are operationalized, leading to our evaluation evidence in Table 1. The logic model (Figure 1) was not only a conceptual framework, but served as a practical guide for tracking activities and outcomes across MW CTR-IN. Our evaluation strategy mapped short- and long-term outcomes from the logic model to specific metrics collected in the custom portal, allowing for real-time assessment and continuous improvement. The data informed annual reviews and mid-cycle adjustments in pilot program design, core coordination, and investigator support. By utilizing current knowledge, our experience provides information and direction to improve the operations of new centers. We believe that this case study technique and expertise we have gathered can help other CTR networks create pilot project programs and portals comparable to this one, which has been shown to increase support for researchers and community partners.

**Table 1.** Illustrates how CP3 core findings (over 10 years), align with the MW CTR-IN logic model, showing how the portal operationalized outcomes and enabled on-going program evaluation and improvement. This alignment validates the logic model as an effective tool for managing and assessing translational research in underserved regions

MW CTR-IN LOGIC MODEL OUTCOMES	PORTAL OPERATIONALIZATION	EVALUATION EVIDENCE
Increase extramural grant submissions and awards	Tracked through follow-on funding reports and investigator Individual Development Plans	42% of CP3-C awardees submitted external grants; 28% were funded
Build collaborative, cross-institutional teams	Monitored through portal-based Co-Principal Investigator and Co-author networks	60% of funded investigators had cross-institutional collaborations
Enhance investigator readiness and professional development	Measured via engagement in PD-C workshops and mentoring participation	120+ ESIs attended PD-C workshops; 38% secured extramural funding
Expand community-engaged research	Documented via Community Engaged Research Pilot Grant applications and CEO-C consultations	14 community-engaged projects funded, 20+ CAB consultations recorded
Strengthen infrastructure and CTR capacity	Captured by portal usage, core service tickets, and internal reporting	1,510 core interactions logged across 558 support tickets; >70 BERD-C consults
Improve program oversight and responsiveness	Achieved through portal automation, quarterly reporting, and core feedback loops	Real-time metrics allowed iterative program improvements and targeted outreach
**ABBREVIATIONS LISTED IN TABLE**		
**BERD-C =** Biostatistics, Epidemiology, Research & Design Core **CAB =** Community Advisory Board **CBPR =** Community-Based Participatory Research **CEO-C =** Community Engagement & Outreach Cor		**CTR =** Clinical & Translational Research **ESI =** Early-Stage Investigator **PD-C =** Professional Development Core
